# Effect of maternal COVID-19 vaccination on mandibular molars development in albino rats offspring

**DOI:** 10.1038/s41598-025-18115-6

**Published:** 2025-09-12

**Authors:** Lama E. Dawoud, Enas M. Hegazy, Elham F. Mahmoud

**Affiliations:** Oral Biology Department, Faculty of Dentistry, Suez Canal University, Ismailia, 41523 Egypt

**Keywords:** Development, COVID-19, Vaccine, Real time PCR, RANKL, OPG, BMP-2, Immunology, Molecular biology, Health care, Medical research

## Abstract

The aim of this study was to investigate the effect of maternal vaccination by coronavirus (COVID-19) vaccine on the development of mandibular first molar of albino rats offspring. Pregnant female rats were divided into control and vaccinated groups injected intramuscularly by 0.035 mL of either saline or AstraZeneca vaccine, respectively. The offspring were examined through serology, histology, histomorphometric and PCR analysis. Vaccinated mothers and their offspring showed mean antibody concentrations of 92.57 AU/mL and 100 AU/mL, respectively. Histologically, both groups were nearly at the same stages of odontogenesis on the third and ninth postnatal days. Higher bone deposition was quantified in the vaccinated group on the third and ninth postnatal days except below the tooth germ on the third day with statistically significant difference (p value ≤ 0.05). Significant increases in expression of Receptor activator of nuclear factor ligand (RANKL) gene (3.53 fold) and bone morphogenic protein-2 (BMP-2) gene (4.76 fold) were noted on the ninth postnatal day. However, osteoprotegrin (OPG) gene was downregulated (0.40 fold) on the ninth postnatal day with statistically significant differences (p value ≤ 0.05) between both groups. We conclude that AstraZeneca vaccine administration during pregnancy is generally safe regarding the teeth development in the offspring.

**Supplementary Information:**

The online version contains supplementary material available at 10.1038/s41598-025-18115-6.

## Introduction

Tooth eruption is crucial for the existence of humans and mammals overall, with direct impact on the growth and maturity of the lower face, mastication for nutrition and energy intake, speech, and aesthetics^1^. It is a physiological process where a tooth is vertically moved from its original non-functional, developmental position towards its functional position, with the purpose of occluding with its opposing in the dental arch^2^.

The dental formula of rats is I 1/1, C 0/0, P 0/0, M 3/3 = 4 teeth in each quadrant. Rats have only one permanent set of teeth. The incisors are open rooted and continuously erupt during their lives, but the molars are permanently rooted and do not continuously erupt. Rat teeth vary morphologically from human teeth, but their enamel and dentin are similar histologically to that of human, which can be highly valuable for investigating the development of human teeth^3^.

Receptor activator of nuclear factor ligand (RANKL) plays a chief role in the growth of lymphoid organs and the development of mammary glands in the time of pregnancy. RANKL-deficient mice experienced a systemic loss of lymph node formation^4^. The action of RANKL and Osteoprotegrin (OPG) is mandatory for tooth eruption; in null mice lacking the RANKL gene, teeth are unerupted^5,6^.

In vivo, expression of OPG is reduced in the dental follicle at day 3 post-natally. The prohibition of gene expression of OPG allows for osteoclast formation, causing a sharp increase in the number of osteoclasts^7^.

The RANKL-RANK-OPG system shows an important biological function in bone homeostasis and bone remodeling^8^. Disturbances of the RANKL-RANK-OPG system at the bone level certainly will lead to bone-related pathologies, for example post-menopausal osteoporosis^9^. Furthermore, The RANKL-RANK-OPG system/pathway is responsible for the organogenesis of the primary and secondary lymphoid tissues, regulates cytokine-receptor interactions through regulation of RANKL expression on developing T cells, which in turn interacts with RANK that is expressed on main lymphoid cells in order to stimulate signaling pathways that affect functions of T cells, mainly antigen presentation. Dysregulation of the RANKL-RANK-OPG system is therefore correlated with T cell intolerance and autoimmune disease^10^.

In rat molars dental follicle, Bone morphogenic protein-2 (BMP-2) is highly expressed in the basal half of the dental follicle than in its coronal half. Chronologically, BMP-2 gene expression starts to increase on day 3 with its maximal expression on day 9 after birth. These expression times are correlated with the initiation of alveolar bone formation at the base of the crypt on the third day postnatally and with rapid bone formation by day 9. Accordingly, BMP-2 may possibly regulate the osteogenesis of the basal bone in the crypt^11^. Expression of BMP-2 is enhanced by the transforming growth factor α (TGF-α) which is highly expressed the dental follicle specially at its basal half to allow alveolar bone formation at the fundus of the crypt^12^.

The current study depends on the hypothesis that RANKL, OPG and BMP-2 have a chronological order when expressed in the dental follicle, mentioned by Wise et al.^7,12^ in order to study the development of the mandibular first molar in the offspring of mothers vaccinated with AstraZeneca vaccine.

SARS-CoV-2 is a new human-infecting beta-coronavirus not previously detected in humans or animals. Various strategies have been adopted by the WHO to protect against spreads of SARS-CoV-2 such as social distancing, travel limitations, and obligatory masks in public areas. However, the most efficient strategy was vaccination. Laboratory and clinical trials have been conducted to prove the safety and efficacy of different vaccines. Several platforms and companies have developed different vaccines of diverse composition among them inactivated, live attenuated, recombinant protein, vectored, and mRNA-based vaccines^13^.

Oxford/AstraZeneca vaccine (Vaxzevria^®^) is a recombinant adenovirus vaccine that uses the S glycoprotein against SARS-CoV-2. It consists of the replication-deficient simian adenovirus vector ChAdOx1, containing the full-length spike protein of SARS-CoV-2, along with a tissue plasminogen activator leader sequence. It employs an altered chimpanzee DNA adenovirus that sends DNA vectors to the cellular cytoplasm which then transfers to the nucleus to allow the production of S protein particles. Next, S protein particles are expressed on the cellular surface membrane via MHC-I and MHC-II complexes which in turn enhance CD8 + T-cells, B-cells and plasma cell stimulation^14^.

In vitro studies reported an increase in activity of CD4 + T cells in production of tumor necrosis factor and interferon-gamma production when stimulated by the viral antigen^15^. In vivo, a single dose of the adenoviral vectored vaccine promoted Nabs and assisted monocyte-mediated and neutrophil-mediated phagocytosis^16^. The AstraZeneca vaccine is believed to be economical and can be readily stored than other vaccines in refrigerators^17^.

Pregnant females with COVID-19 are at higher risk of hospital admission to an intensive care unit and preterm delivery compared to healthy pregnant females. The outcome worsens in case of infection at early gestational age, advanced maternal age, increased maternal body mass index, and low neonatal birthweight. Therefore, pregnant females should protect themselves through proper social distancing, good hand hygiene, face coverings, and vaccination^18^.

Unfortunately, some adverse pregnancy consequences may occur rarely following vaccination such as preeclampsia, cesarean delivery, postpartum hemorrhage, and abortion. Therefore, vaccine hesitancy among pregnant females is still present and many studies are still needed to confirm the overall safety of COVID-19 vaccines administration during pregnancy concerning the course of pregnancy and the development of the offspring^19^. It has been reported that mRNA COVID-19 vaccines are safer than adenoviral vector and inactivated vaccines concerning tooth and palatal development^20^.

The purpose of the present study is to answer the question of “ what is the probable effect of maternal COVID-19 vaccination on the development and eruption of the first mandibular molar in albino rat offspring?”. The null hypothesis is there is no significant effect of vaccination during pregnancy on the development and eruption of the first mandibular molar in the offspring.

## Materials and methods

### Ethical considerations

The present research has been conducted after the approval of the Research Ethics Committee (REC) of the Faculty of Dentistry, Suez Canal University (approval number: 510/2022). All procedures were performed in compliance with ARRIVE guidelines (PLoS Bio 8(6), e1000412, 2010). Moreover, every technique was used in accordance with the applicable rules and regulations.

### Animals and mating

Animals were obtained and housed in the animal house of Faculty of Medicine, Suez Canal University. This study was conducted using adult female albino rats, with body weight 160–180 gm at the start of the experiment. Rats were paired for mating such as each female rat was housed 1:1 with a non-treated male until mating is confirmed by the presence of a vaginal plug and the identification of male sperms in vaginal smears in 1–3 days^21^. Day of confirmation of pregnancy was considered as the first gestational day.

Following evidence of mating, the females were separately housed through gestation and lactation in well-ventilated animal house. All animals were fed with an adequate natural diet consisting of fresh vegetables, dried bread and given drinking water ad libitum. They were kept under proper conditions of temperature and a 12-h light /12-h dark cycle.

### Animal grouping and study procedure

Pregnant female rats were randomly and equally divided into two groups using an online randomization tool (https://www.sealedenvelope.com/randomisation/) according to sample size calculation mentioned later.

1. Group I (Control group): received saline solution by injection intramuscularly in the thigh muscle of each hindlimb. The amount of saline solution was 0.035 mL injected in each hindlimb (total dose per session: 0.07 mL/session).
2. Group II (Vaccinated group): received COVID-19 vaccine AstraZeneca (ChAdOx1-S recombinant, AstraZeneca, UK) (batch PW40179) as the test agent. Pregnant female rats were injected by 0.035 mL of vaccine intramuscularly in the thigh muscle of each hindlimb (total dose per session: 0.07 mL/session)^18^.

### Treatment protocol

Dams were injected with AstraZeneca vaccine or saline on gestational days 6 and 15, the same protocol adopted by a non-clinical trial on pregnant rats^18^. Dams were allowed to deliver normally after nearly 21–23 days with regular observation to check for any difficulties or abnormalities during parturition. Average number of offspring born for each pregnant female rat was 5–7. The offspring were euthanized by cervical dislocation on the third and ninth postnatal days (PNDs). Male albino rats used in this study were not treated and were excluded from the study after confirmation of mating and pregnancy in female rats.

### Methods of evaluation

#### Physical examination

Mortality of pregnant rats and later the offspring were checked twice daily during the study, one time at the start and the other time at the end of the working day. The response of pregnant female rats to vaccine were observed and compared to the control group particularly during the first two hours after injection. Weight of pregnant mothers was checked on the 1st, 5th and 16th day of pregnancy. Weight of the offspring was checked on postnatal days 0, 3, 9, 14 and 30 to check for normal growth and development.

#### Immunogenicity examination

Blood samples were obtained at particular times for serological immunogenicity testing. Relative quantification of anti-spike glycoprotein antibodies in rat serum was performed using electro chemiluminescent immunoassay. The methodology was the same as it was used by Stebbings et al.^18^. Data were reported in arbitrary units/mL (AU/mL) relative to the calibrator.

Approximately 0.25 mL of blood was collected from the orbital vein, pregnant dams had samples collected on GD 15 (second dose day). Offspring samples, pooled per litter, were also collected on PND 21^18^.

#### Histological evaluation

At the proposed time (third and ninth postnatal days), the offspring were euthanized, and the mandibles of each rat were dissected out. Then the samples were fixed immediately in a 10% buffered formalin solution for 48 h. Decalcification of mandibles is carried out using 10% EDTA solution (PH 7.4) until tissue is properly softened. One half of the mandible is used for histological evaluation and the other half is used for molecular investigation. Specimens were processed and stained with Hematoxylin and Eosin stains to assess the mandibular first molar tooth germ developmental stage.

#### Histo-morphometric evaluation

Histomorphometric evaluation was carried out at Faculty of Medicine, Suez Canal University. Six high power fields (× 200) were selected for evaluation in each serial section of the studied groups. Area percentage was determined via Leica QWin 500 image analyzer computer system (England). This image analyzer entails a Leica microscope, a colored video camera, colored monitor and a hard disc of a Leica IBM personal computer linked to the microscope and managed by Leica QWin 500 software. Records of each parameter were statistically described in terms of mean and standard deviation (mean ± SD) for area percentage. Area percentage (Fig. S1) was determined for bone deposition directly above and beneath the tooth germ (on the superior and inferior areas of the crypt) on the third and ninth postnatal days according to the standardized methodology proposed by^22^.

#### Molecular investigation using real time PCR

All procedures of molecular investigation were performed at Faculty of Veterinary Medicine, Kafr El sheikh university.

*RNA extraction* Pure RNA was extracted using a total RNA Purification Kit following the manufacturer protocol (Thermo Scientific, Fermentas, K0731). Pure RNA is eluted under low ionic strength conditions with nuclease-free water.

*cDNA synthesis* This technique used reverse transcription kits (Thermo Scientific™, Fermentas, #EP0451) to make a DNA (cDNA) copy of the RNA. The kits particularly employed RevertAid™ H Minus Reverse Transcriptase enzyme for this reaction. The mixture was incubated for 60 min at 42 °C. The reaction was terminated by heating at 70 °C for 10 min.

*Real time PCR* Real-time PCR with SYBR Green was used to measure expression of mRNAs, with β-actin as an internal reference. The isolated cDNA was amplified using 2X Maxima SYBR Green/ROX qPCR Master Mix following the manufacturer protocol (Thermo scientific, USA, K0221) and gene specific primers. The web-based tool, Primer 3 was used to design these primers based on published rat sequences. To ensure primer sequence is unique for the template sequence; we checked similarity to other known sequences with basic local alignment search tool (BLAST)^23^. The final reaction mixture was put in a PikoReal™ 24 real-time PCR system (Thermo Scientific™, USA).

The quantities critical threshold (Ct) of target gene were normalized with quantities (Ct) of housekeeping gene (*ß-actin*) by using the 2^−∆∆Ct^ method^24^ as follow:

- The control group was used as calibrator, while other groups represented as test groups in both target and reference gene.
- The critical threshold cycle numbers (Ct) of target gene were normalized to that of reference (ref.) gene, in both the test groups and the control group by using the following equations:
  $$\Delta {\text{Ct }}\left( {{\text{test}}} \right)={\text{Ct }}\left( {{\text{target in test groups}}} \right) -{\text{Ct }}\left( {{\text{ref}}.{\text{ in test groups}}} \right)$$
  $$\Delta {\text{Ct }}\left( {{\text{calibrator}}} \right)={\text{Ct }}\left( {{\text{target in control}}} \right) -{\text{ Ct }}\left( {{\text{ref}}.{\text{ in control}}} \right)$$
- The ∆Ct of the test genes were normalized to the ∆Ct of the calibrator:
  $$\Delta \Delta {\text{Ct}}=\Delta {\text{Ct }}\left( {{\text{test}}} \right) - \Delta {\text{Ct }}\left( {{\text{calibrator}}} \right)$$
- Fold change of relative gene expression was calculated as follow:
  $${\text{Fold change }}={\text{ }}\left( {{{\text{2}}^{ - \Delta \Delta {\text{Ct}}}}} \right).$$

#### Statistical analysis

- Sample size calculation

The sample size for this study was calculated using the G-Power statistical power analysis program (version 3.1.9.7)^25^.

$${\text{Total sample size N}}=\frac{{{{\left( {1.96} \right)}^2} \times {{\left( {2.88} \right)}^2}}}{{{{\left( 2 \right)}^2}}}={\text{ 7}}.{\text{99}} \approx {\text{ 1}}0\left( {{\text{pregnant rats}}} \right).$$

- Statistical tests

Data were examined for normality via Kolmogorov-Smirnov test of normality. The outcomes of this test pointed out that some data were normally distributed (parametric data), accordingly descriptive analysis, One Way-Anova, Post Hock tests were operated for intergroup relationship and others undergo independent sample t test and Levene’s Test. Statistical analysis was conducted with SPSS 26.0 (Statistical Package for Scientific Studies, SPSS, Inc., Chicago, IL, USA) for Windows.

## Results

### Results of physical examination

**Fig. 1:**
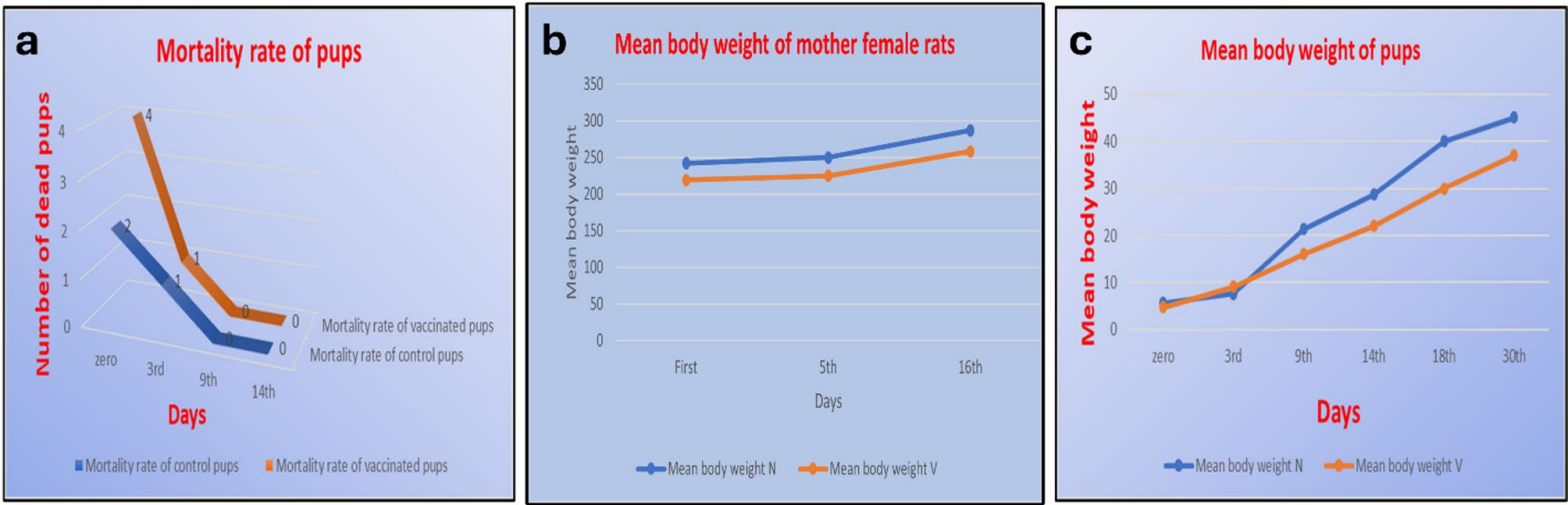
**a** Line graph showing the mortality rate of control and vaccinated offspring on 0, 3rd, 9th and 14th postnatal days. **b** Line graph showing the mean weight gain of mother rats during the gestation period. **c** Line graph showing the mean weight gain of offspring from birth to one month postnatal.

*Mortality rate* Data revealed that there is no significant difference between mortality rate of offspring of control and vaccinated groups at p value ≥ 0.05 (Fig. 1a).

*Body weight assessment* All through the experiment, there was no inflammation at the site of injection on any dosing time, no effects on maternal body weight gain, or food consumption. Data revealed that there was no significant difference between mother body weight of control and vaccinated groups at p value ≥ 0.05. Moreover, there was no significant difference between offspring body weight of control and vaccinated groups at p value ≥ 0.05 (Fig. 1b, c).

### Results of immunogenicity testing and seroconversion

Immunogenicity testing of vaccinated mothers on GD 15 suggested an antibody response to spike glycoprotein, with seroconversion (change from seronegative to seropositive) of all vaccinated mothers (mean [SD] antibody concentration: 92.57 AU/mL). Samples collected from offspring on PND 21 were all seropositive (100 AU/mL).

### Results of histological examination

**Fig. 2:**
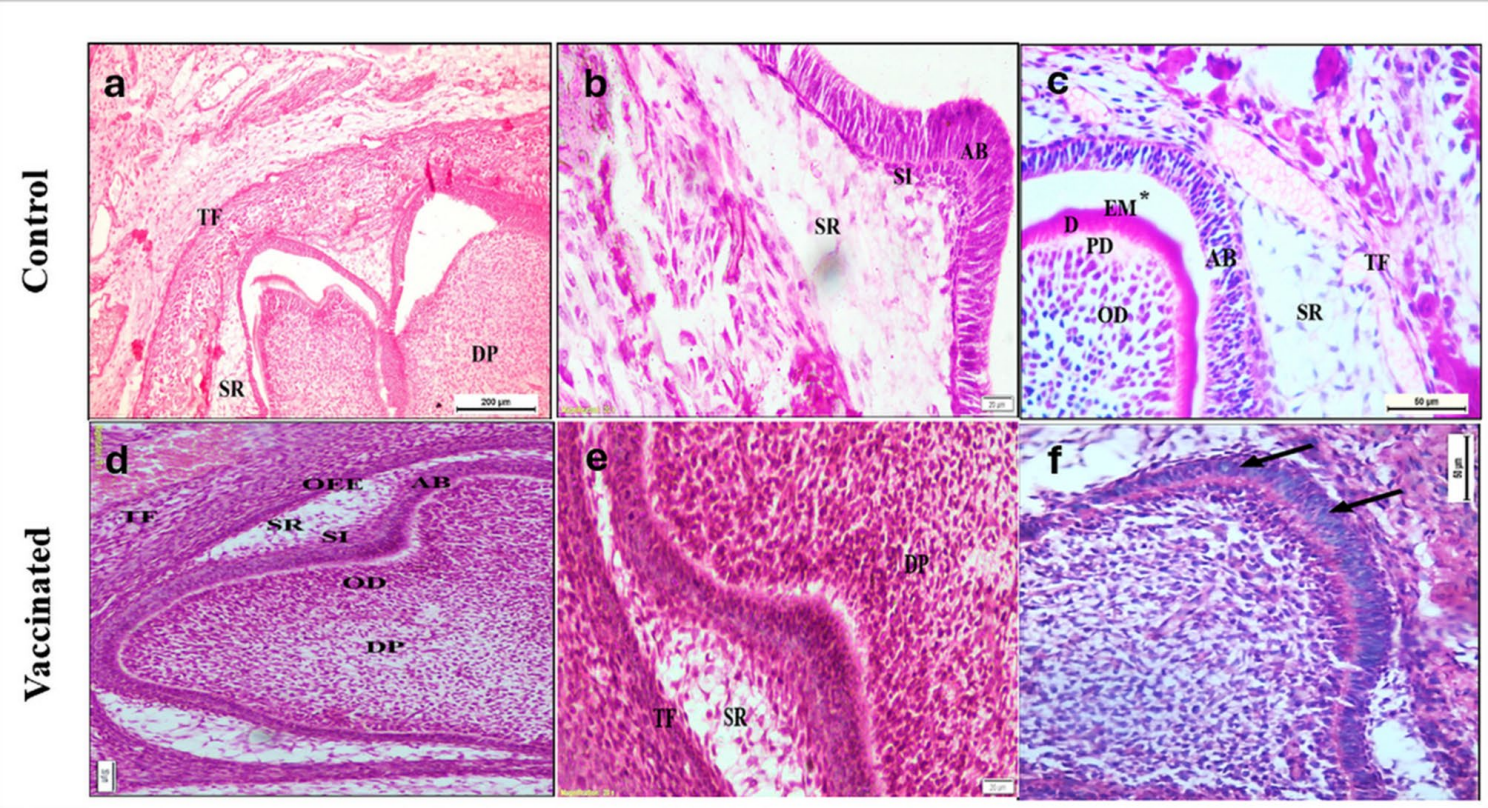
Photomicrographs showing the developing lower first molar enamel organ of control group offspring (**a**–**c**) and vaccinated group offspring (**d**–**f**) on the third postnatal day at early bell stage or transition from early to advanced bell stages of odontogenesis. Control group: **a** Enamel organ at the transition between early and late bell stages of odontogenesis with nearly no hard tissue formation. Stellate reticulum cells (SR) seen as star shaped cells overlying the preameloblasts. Note the highly cellular dental papilla (DP) and tooth follicle (TF) (mag. × 100, scale bar 200 μm). **b** Enamel organ at the early bell stage of odontogenesis. Stellate reticulum cells (SR) seen as star shaped like cells. Stratum intermedium cells (SI) also evident as 2–3 layers of flat cells overlying the tall columnar ameloblasts (AB) (mag. × 200, scale bar 20 μm). **c** Note the tall columnar ameloblasts (AB) with their nuclei still not at the same level and a thin layer of enamel matrix (EM). Odontoblasts (OD) seen lining the dental papilla with apparent pseudostratification and a thin layer of predentin (PD) and dentin (D). the white space (*) is an artifact (mag. X400, scale bar: 50 μm). Vaccinated group: **d** Enamel organ at the transition between early and advanced bell stages of odontogenesis. Note the stellate reticulum (SR), the outer enamel epithelium (OEE), ameloblasts (AB), and stratum intermedium (SI). Also note the odontoblastic layer (OD), dental papilla (DP) and the tooth follicle (TF) (mag. × 100, scale bar: 50 μm). **e** Same stage as **d** (mag. × 200, scale bar: 20 μm). **f** Enamel organ at the advanced bell stage of odontogenesis. Nuclei of ameloblasts still not aligned at the same level (arrows) (mag. × 400, scale bar: 50 μm).

*Third postnatal day* The lower first molar tooth germ of the offspring of control mothers showed transition between early bell stage and advanced bell stage. The ameloblasts appeared as tall columnar cells with elongated deeply stained nuclei. Their nuclei were still not at the same level. Thin layers of hard tissues (enamel matrix, dentin and predentin) were seen in certain areas of the tooth germ. The cervical loop started to proliferate apically to form the epithelial root sheath of Hertwig (HERS) (Fig. 2a–c). The teeth germ of the lower first molar of vaccinated offspring showed also transition between early and advanced bell stage with some areas of hard tissue formation (Fig. 2d–f).

**Fig. 3:**
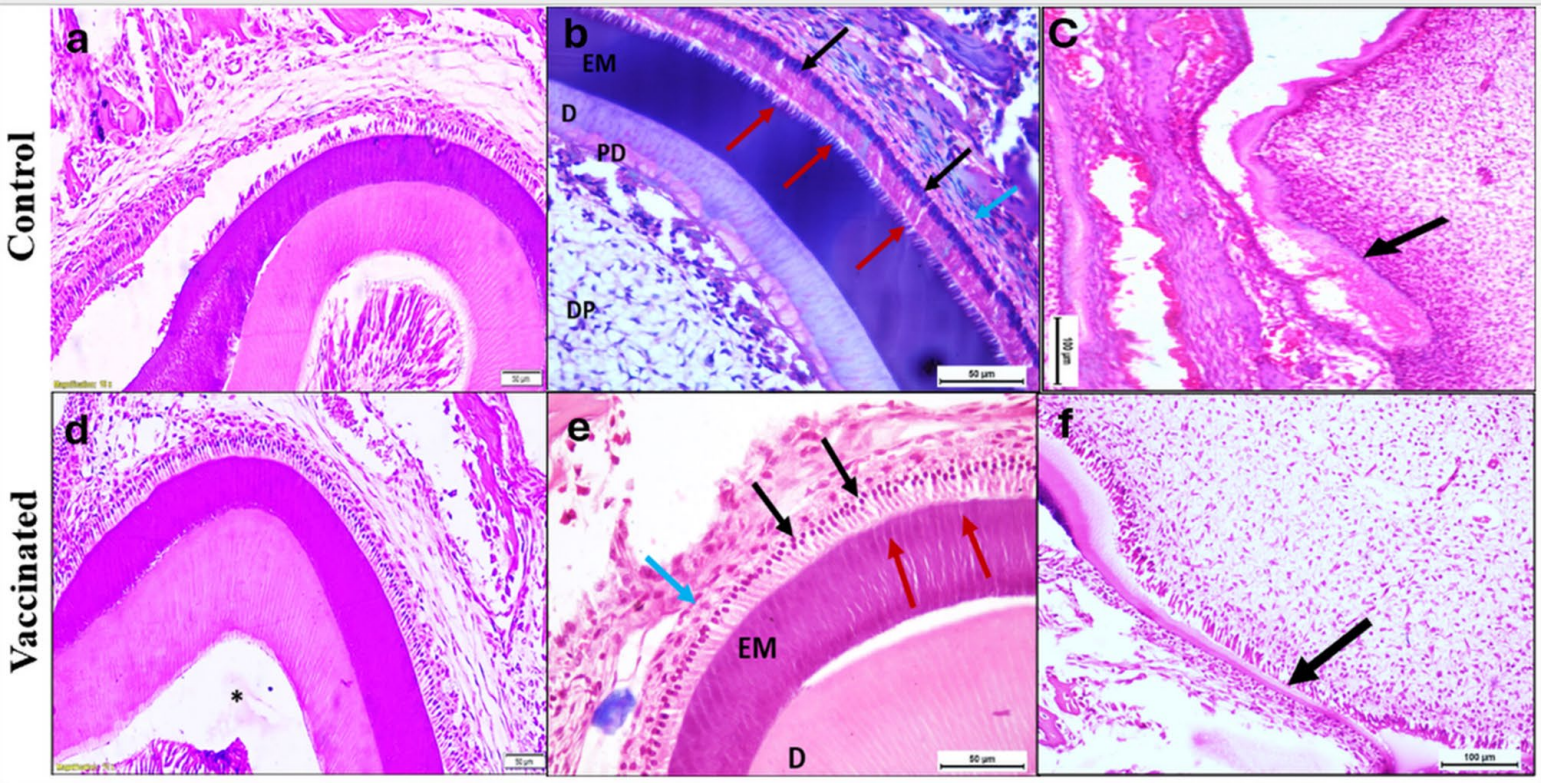
Photomicrographs showing the developing lower first molar enamel organ of control group offspring (**a**–**c**) and vaccinated group offspring (**d**–**f**) on the ninth postnatal day at the advanced bell stage. Control group: **a** Enamel organ showing considerable hard tissue formation (mag. × 100, scale bar: 50 μm). **b** Hard tissue formation (EM: enamel matrix, D: dentin, PD: predentin). Note the tall columnar secretory ameloblasts having proximal nuclei (black arrows) and Tome’s processes at the distal ends inserted into the enamel matrix giving saw tooth appearance (red arrows). Also note the stratum intermedium (blue arrow) overlying the ameloblasts and the highly cellular dental papilla (DP) (mag. × 400, scale bar: 50 μm). **c** The cervical loop region (arrow) of the lower first molar tooth germ (mag. × 200, scale bar: 100 μm). Vaccinated group: **d** Enamel organ in the advanced bell stage of odontogenesis. The white space (*) is an artifact (mag. × 100, scale bar: 50 μm). **e** Hard tissue formation (EM: enamel matrix, D: dentin) in developing lower first molar enamel organ. Note the tall columnar secretory ameloblasts having proximal nuclei (black arrows) and Tome’s processes at the distal ends inserted into the enamel matrix giving saw tooth appearance (red arrows). Note the 2–3 cell layers of stratum intermedium over the ameloblasts (blue arrow) (× 400, scale bar: 50 μm). **f** The cervical loop region of the lower first molar tooth germ. Note the formation of root predentin and dentin (arrow) (mag. × 200, scale bar: 100 μm).

*Ninth postnatal day* The lower first molar tooth germ of control group offspring showed complete crown formation in advanced bell stage with considerable hard tissue (predentin, dentin and enamel matrix) formation. The ameloblasts were clearly seen with their processes having the saw tooth appearance and their nuclei arranged at the same level at the proximal side of the cell. The cervical loop was completely formed with formation of HERS and proliferation of odontoblasts to form the root dentin (Fig. 3a–c). The vaccinated group showed their first molar teeth germ also at the advanced bell stage with similar histological features (Fig. 3d–f).

### Results of histomorphometric analysis of area of bone trabeculae above and below the tooth germ

**Fig. 4:**
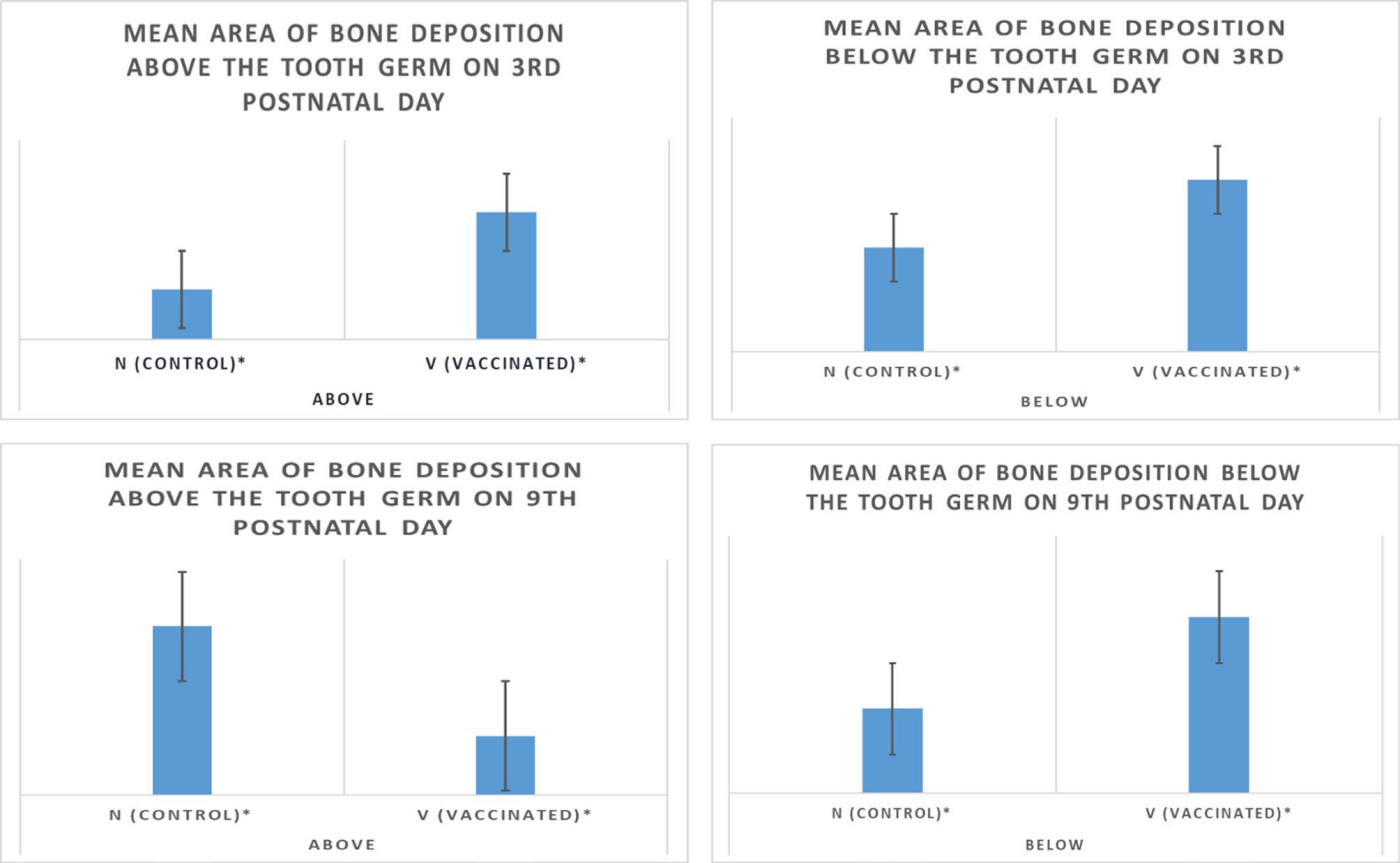
Graphical presentation of the results of histomorphometric analysis showing values of mean area of bone deposition on the third and ninth postnatal days above and below the developing tooth germ of control (N) and vaccinated (V) groups. Asterisks indicate statistically significant differences.

*Third postnatal day* Data revealed that there was a high significant difference between areas of bone deposition in the form of bone trabeculae above the tooth germ (coronally) in control group offspring and above the tooth germ in vaccinated group offspring on the third postnatal day at p value ≤ 0.05. Furthermore, data showed a high significant difference between areas of bone deposition in the form of bone trabeculae below the tooth germ (apically) in control group offspring and below the tooth germ in vaccinated group offspring on the third postnatal day at p value ≤ 0.05 (Fig. 4; Table 1).

**Table 1:** Statistical results for the bone area on the third and ninth postnatal days above and below the developing tooth germ of control and vaccinated groups.

Histomorphometric analysis					
Tukey HSD^a^					
Groups	Number of samples	Subset for alpha = 0.05			
		1	2	3	4
Control-third day above	6	2.991167			
Vaccinated-third day above	6		7.667667		
Control-third day below	6			11.157000	
Vaccinated-third day below	6				18.392333
Significance		1.000	1.000	1.000	1.000
Control-ninth day-above	6		7.162833		
Vaccinated-ninth day-above	6	2.517000			
Control-ninth day-below	6			11.454833	
Vaccinated-ninth day-below	6				23.906167
Significance		1.000	1.000	1.000	1.000

^a^Means for groups in homogeneous subsets are displayed. Uses Harmonic Mean Sample Size = 6.000.

*Ninth postnatal day* Data revealed that there was a high significant difference between areas of bone deposition in the form of bone trabeculae above the tooth germ (coronally) in control group offspring and above the tooth germ in vaccinated group offspring on the ninth postnatal day at p value ≤ 0.05. Furthermore, data showed a high significant difference between areas of bone deposition in the form of bone trabeculae below the tooth germ (apically) in control group offspring and below the tooth germ in vaccinated group offspring on the ninth postnatal day at p value ≤ 0.05 (Fig. 4; Table 1).

### Results of molecular investigations (RT-qPCR)

**Fig. 5:**
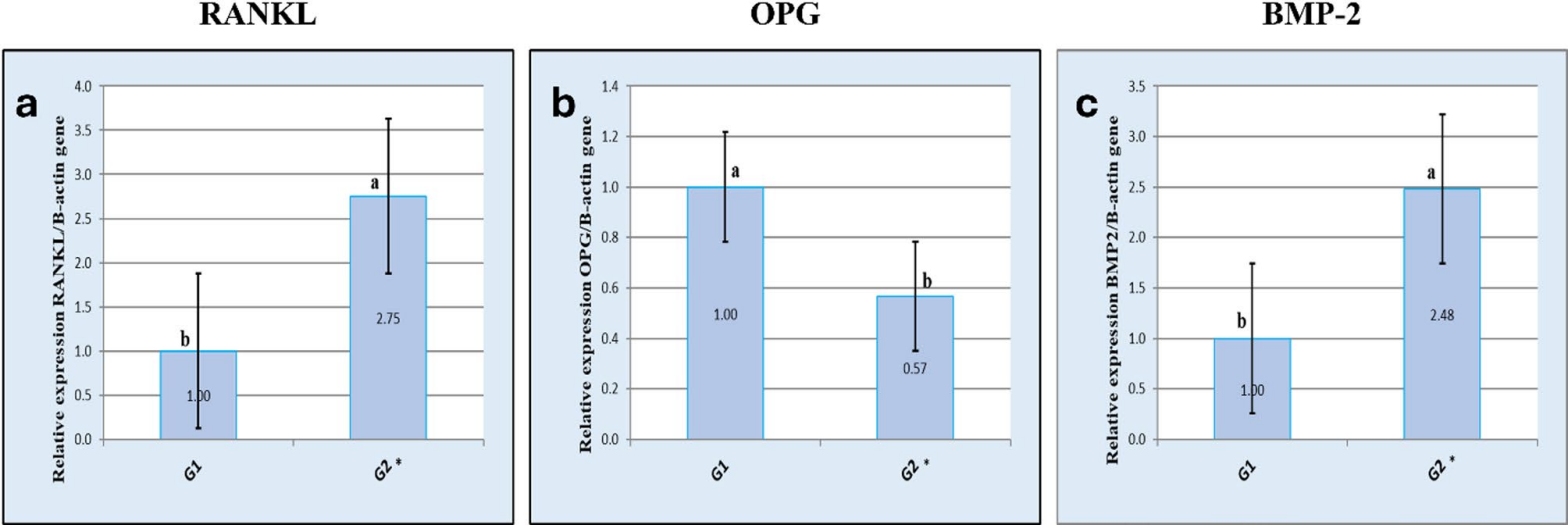
Results of real-time quantitative PCR analysis of gene expression on the third postnatal day. **a** Graphical presentation of real-time quantitative PCR analysis of the expression of RANKL on the third postnatal day. **b** Graphical presentation of real-time quantitative PCR analysis of the expression of OPG on the third postnatal day. **c**: Graphical presentation of real-time quantitative PCR analysis of the expression of BMP2 on the third postnatal day. Asterisks indicate statistically significant differences.

**Fig. 6:**
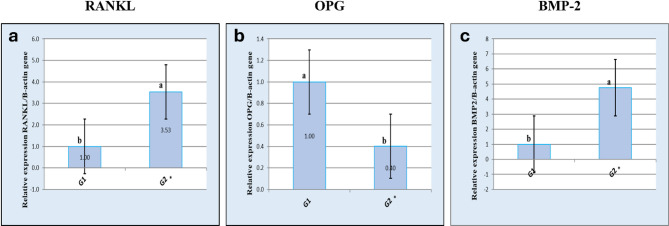
Results of real-time quantitative PCR analysis of gene expression on the ninth postnatal day. **a** Graphical presentation of real-time quantitative PCR analysis of the expression of RANKL on the ninth postnatal day. **b** Graphical presentation of real-time quantitative PCR analysis of the expression of OPG on the ninth postnatal day. **c** Graphical presentation of real-time quantitative PCR analysis of the expression of BMP2 on the ninth postnatal day. Asterisks indicate statistically significant differences.

*Results of real-time quantitative PCR analysis of the expression of RANKL gene* Real time quantitative PCR analysis of RANKL gene in the vaccinated group on the third and ninth postnatal days showed 2.75 and 3.53 fold changes respectively which indicated higher expression of RANKL gene in the vaccinated group over the control group on both days with statistically significant difference between the two groups (Figs. 5a and 6a; Tables 2 and 3).

**Table 2:** Calculations of fold change for the relative expressions of the RANKL, OPG and BMP-2 genes on the third postnatal day.

Gene	Group	Gene Aver CT	Delta Ct	Delta delta Ct	Fold change	SEM
RANKL	Control third day	21.84	− 2.55	0.00	1.00	0
	Vaccinated third day	20.42	− 4.01	− 1.46	2.75	0.12
OPG	Control third day	23.3	− 1.09	0.00	1.00	0
	Vaccinated third day	24.06	− 0.27	0.82	0.57	0.02
BMP-2	Control third day	23.3	− 1.09	0.00	1.00	0
	Vaccinated third day	21.93	− 2.40	− 1.31	2.48	0.14

**Table 3:** Calculations of fold change for the relative expressions of the RANKL, OPG and BMP-2 genes on the ninth postnatal day.

Gene	Group	Gene Aver CT	Delta Ct	Delta delta Ct	Fold change	SEM
RANKL	Control ninth day	22.85	− 1.54	0.00	1.00	0
	Vaccinated ninth day	21.47	− 3.36	− 1.82	3.53	0.15
OPG	Control third day	23.15	− 1.24	0.00	1.00	0
	Vaccinated ninth day	24.2	0.07	1.31	0.40	0.02
BMP-2	Control ninth day	22.1	− 2.29	0.00	1.00	0
	Vaccinated ninth day	20.19	− 4.54	− 2.25	4.76	0.23

*Results of real-time quantitative PCR analysis of the expression of OPG gene* Real time quantitative PCR analysis of OPG gene in the vaccinated group on the third and ninth postnatal days showed 0.57 and 0.40 fold changes respectively which indicated reduced expression of OPG gene in the vaccinated group over the control group on both days with statistically significant difference between the two groups (Figs. 5b and 6b; Tables 2 and 3).

*Results of real-time quantitative PCR analysis of the expression of BMP2 gene* Real time quantitative PCR analysis of BMP-2 gene in the vaccinated group on the third and ninth postnatal days showed 2.48 and 4.76 fold changes respectively which indicated increased expression of BMP-2 gene in the vaccinated group over the control group on both days with statistically significant difference between the two groups (Figs. 5c and 6c; Tables 2 and 3).

## Discussion

Pregnant females were specially targeted by the COVID-19 vaccination due to their higher susceptibility and compromised immunity. Therefore, the safety of vaccination must be meticulously investigated in all fields concerning the development of the fetus or the health of the mother^26^. In the current study, we investigated the effect of maternal COVID-19 vaccination using the AstraZeneca vaccine on the development and eruption of mandibular first molar of the rat offspring.

The albino rat is a suitable model for examining the eruption process, especially the rat molars, which are highly comparable to human molars^27^. Rats have been found to be excellent for examining the growth, development of oral tissues in the offspring due to their short gestation period (21–22 days)^28,29^.

The AstraZeneca vaccine, also known as Oxford, and ChAdOx1 nCov-19 vaccine is a commonly used vaccine against SARS-COV-2. Vaccine was administered intramuscularly using the same methodology of Stebbings et al.^18^ utilizing a vaccine dose that exceeds the human dose on a mg/kg base. However, other routes of administration have been employed by other authors such as retrograde instillation of COVID-19 vaccine into the submandibular salivary gland duct. This route has been proved to be more effective in production of anti-COVID-19 IgG levels than the intramuscular one^30^.

In the present study, AstraZeneca vaccine resulted in an increase of anti-spike glycoprotein antibodies in females and offspring serum. This result agreed with other studies using the same vaccine on experimental animals^18^ and humans^16^.

In the current study, serum samples collected from offspring on PND 21 were seropositive. Therefore, exposure of offspring to maternal antibodies during lactation is suggested. This is in agreement with the findings of Stebbings et al.^18^. This exposure to maternal antibodies suggests a protective shield for the nursing infant through the presence of anti-SARS-CoV-2 antibodies and the complement system activation^31^.

The developmental stage of the mandibular first molar tooth germ in the offspring was captured and assessed at two different timepoints, third and ninth postnatal days and were nearly similar with few histological differences between them.

Normally, the rat mandibular first molar eruption occurs on the 18th postnatal day^32^. This was evident in the current experiment where both groups showed the same timing of eruption with no detected differences in the shape or position of the erupted molar (Fig. S2).

In a comparable study, female mice were immunized with COVID-19 vaccine (CoronaVac) and mated for pregnancy with un-immunized male mice. The effects of CoronaVac on pregnancy, lactation, and the growth of offspring were investigated. CoronaVac has no effect on tooth eruption which is in accordance to our results^33^ .

Expression of RANKL and OPG is mandatory for tooth eruption^34^. RANKL is maximally expressed on day 9–10 of birth while OPG levels are highly reduced at day 3 of birth in a way that promotes osteoclastogenesis. Therefore, the RANKL/OPG ratio has to be convenient to promote tooth eruption^35^.

In the rat first mandibular molar, on the third postnatal day (primary burst of osteoclastogenesis), increased levels of colony-stimulating factor-1 in the dental follicle decrease OPG levels in a way that the RANKL/OPG ratio allows the maximal osteoclastogenesis that occur at this time. On day 9–10 (secondary burst of osteoclastogenesis), RANKL expression is increased, possibly by the increased tumor necrosis factor-*α*, in order to create a RANKL/OPG ratio, suitable for bone resorption^35^.

In the current study, on the third postnatal day, the expression of RANKL has upregulated (doubled) in the vaccinated group more than the control group. Furthermore, on the ninth day, RANKL expression has been further regulated. These molecular events yield changes in the histomorphometric measurements. On the third day, a boost of bone resorption has been noticed above the developing tooth germ in the vaccinated group when compared to the control group. Moreover, on the ninth day, similar differences in bone resorptive activity were detected.

Nevertheless, on the third postnatal day, OPG expression has been downregulated in the vaccinated group more than the control group. On day 9, this downregulation has been more marked with further decrease in the expression of OPG in the vaccinated group more than the control group. The decrease in OPG expression is linked to active osteoclastic activity that normally occurs in the primary and secondary bursts of osteoclastogenesis.

The prohibition of OPG gene expression permits the formation and activation of osteoclasts. In vitro studies supported that both colony-stimulating factor-1 and parathyroid hormone-related protein diminished OPG gene expression in the dental follicle cells, proposing that these molecules are involved in the in vivo downregulation of OPG expression^34^.

Bone morphogenic proteins secreted by the dental follicle cells facilitate the transformation of undifferentiated mesenchymal cells into osteoblasts. BMP-2 is responsible for bone deposition under the mandibular first molar tooth germ (in the base of the bony crypt) to enhance the eruption process. It is expressed at higher levels at the basal half of the dental follicle compared to the coronal half of the dental follicle at days 3,5,9 and 11^32,35^. BMP-2 secretion is stimulated and regionally controlled by the tumor necrosis factor alpha (TNF-α) which has been found to be highly expressed in the dental follicle on day 3 and maximally expressed on day 9^36^.

In the current research, BMP-2 expression has dramatically increased in vaccinated group in comparison to control group and also from day 3 to day 9 (doubled) in the vaccinated group. This increase is matched to an increase in areas of bone deposition in the form of bone trabeculae under the developing tooth germ when quantified histomorphologically.

Therefore, in the present study, the overall eruptive process has been boosted in the vaccinated group when correlated with the control group. However, this acceleration has not continued for longer time after day 9 because when examined clinically, the mandibular lower first molar erupted normally at the same time (at day 18) in both groups. This may be explained here by a compensatory or a threshold mechanism for the upregulated genes to return to their normal levels after the ninth day. The mechanism is still unknown, and further studies are needed to emphasize the exact process.

Vaccines are well known to induce the immune system to secrete pro-inflammatory cytokines such as IFNα, TNFα, TGFβ, etc. and may even provoke what is called “cytokine storm”^37^. RANKL is known to be a member of the TNF family and is secreted by activated T lymphocytes. While OPG levels are maintained through the balance with RANK/ RANKL levels. Increased levels of RANKL will stimulate osteoclastogenesis and inhibit the function of OPG^38^. Therefore, vaccination-induced cytokine release is tightly associated with activation of the OPG/RANKL pathway in the immune systems, which, in turn, enhances osteoclastogenesis^39,40^.

Other mechanisms that may contribute to the upregulation of RANKL is that vaccination is known to increase the expression of genes whose products are involved in viral sensing via MHC Class I presentation, interferon signaling, IL-6, and the NFκβ (RANK receptor) signaling pathway. These genes are similarly known to be stimulated in innate cells (such as macrophages and dendritic cells) during viral infections^41^.

Oxidative stress and viral infections have the ability to prompt NF-κB activation. Activation of this pathway responds to distinct stimulus including ligands of tumor necrosis factor receptor (TNFR) superfamily members such as receptor activator of NF-κB (RANK). Therefore, RANKL is increased following activation of NFκβ signaling pathway through binding with RANK receptor mediating osteoclastogenesis^42^.

The increase in BMP-2 expression following vaccination is questionable and may be explained by the increase in cytokines levels produced by activated T cells and macrophages, the same process that occur during inflammation^43^. In fact, COVID-19 vaccination provokes a local and systemic inflammatory response^44^. BMP-2 is known to be a part of the TGFβ family. Moreover, macrophages-secreted cytokines promote bone regeneration through differentiation of mesenchymal stem cells into osteoblasts and related secretion of BMP-2^45^.

Furthermore, TGF-β Signaling is claimed to be increased and promoted in COVID-19 patients. In other words, SARS-CoV-2 can promote expression of TGF-β family genes in blood monocytes and BMP-2 is indeed a part of TGF-β family^46^.

Mature B cells differentiate into immunoglobulin (lg)-secreting plasma cells that in turn secrete antibodies or immunoglobulins. This process occurs upon immune system activation in response to COVID vaccination^47^. Activated B cells produce RANKL and other cytokines that are implied in bone resorption and bone deposition (bone remodeling). Nevertheless, memory B cells could express RANKL on its own^9^.

Surprisingly, both T cells and B cells may have a role in the preservation of bone mass in vivo and B cells may be defined as major producers of OPG. The crosstalk between B-cell and T-cell could control production of bone-related cytokines, since B cells restrain osteoclastogenesis upon activation by T helper 1 cytokines while promoting osteoclastogenesis upon stimulation with T helper 2 cytokines^48^. Moreover, antibodies secreted by B cells may enhance bone preservation and limit bone resorption in vivo^49,50^. Whether this mechanism is related to the upregulated bone morphogenic protein or not is unknown. However, no acceleration of eruption was noted despite the increase of BMP-2 expression, the increased levels of BMP-2 in the vaccinated group may be settled to normal levels after the ninth day. Lack of information about the gene levels after the ninth day may be considered a limitation of this study.

Some limitations have been encountered in the current study such as the necessity of micro-computed tomography (µCT) imaging to capture three-dimensional changes in bone morphology and eruption trajectory and to precisely assess the eruption process through multiple timepoints. Moreover, the need for precise regional quantification of eruption genes through inoculation of the dental follicle at different developmental stages under surgical microscope and the difficult handling of very small animals were considered as limitations of the present study.

Up to our knowledge, this is the first study evaluating the effect of maternal vaccination using AstraZeneca vaccine on the development of the first mandibular molar in albino rat offspring using RT-PCR. Nonetheless, further research is needed to clearly emphasize the effect of vaccine on the unique bone remodeling process that occur during teeth eruption.

## Conclusion

Maternal administration of the AstraZeneca vaccine during pregnancy does not adversely affect the timing or morphology of mandibular first molar development in rat offspring. Moreover, the offspring have been exposed to maternal antibodies during lactation. However, molecular and histomorphometric differences were observed. All these findings support the general safety of AstraZeneca vaccination during pregnancy.

## Supplementary Information

Below is the link to the electronic supplementary material.

Supplementary Material 1

## Data Availability

Data analyzed and used during the current study are available from the corresponding author on reasonable request.
