# Supplementary material for: Effect of maternal COVID-19 vaccination on mandibular molars development in albino rats offspring
Source: Sci Rep. 2025 Sep 12;15:32453. doi: 10.1038/s41598-025-18115-6 (PMC12432186; doi:10.1038/s41598-025-18115-6)
Supplement: Supplementary file 1 — Supplementary Material 1 [file 41598_2025_18115_MOESM1_ESM.docx]

**Supplementary information:**


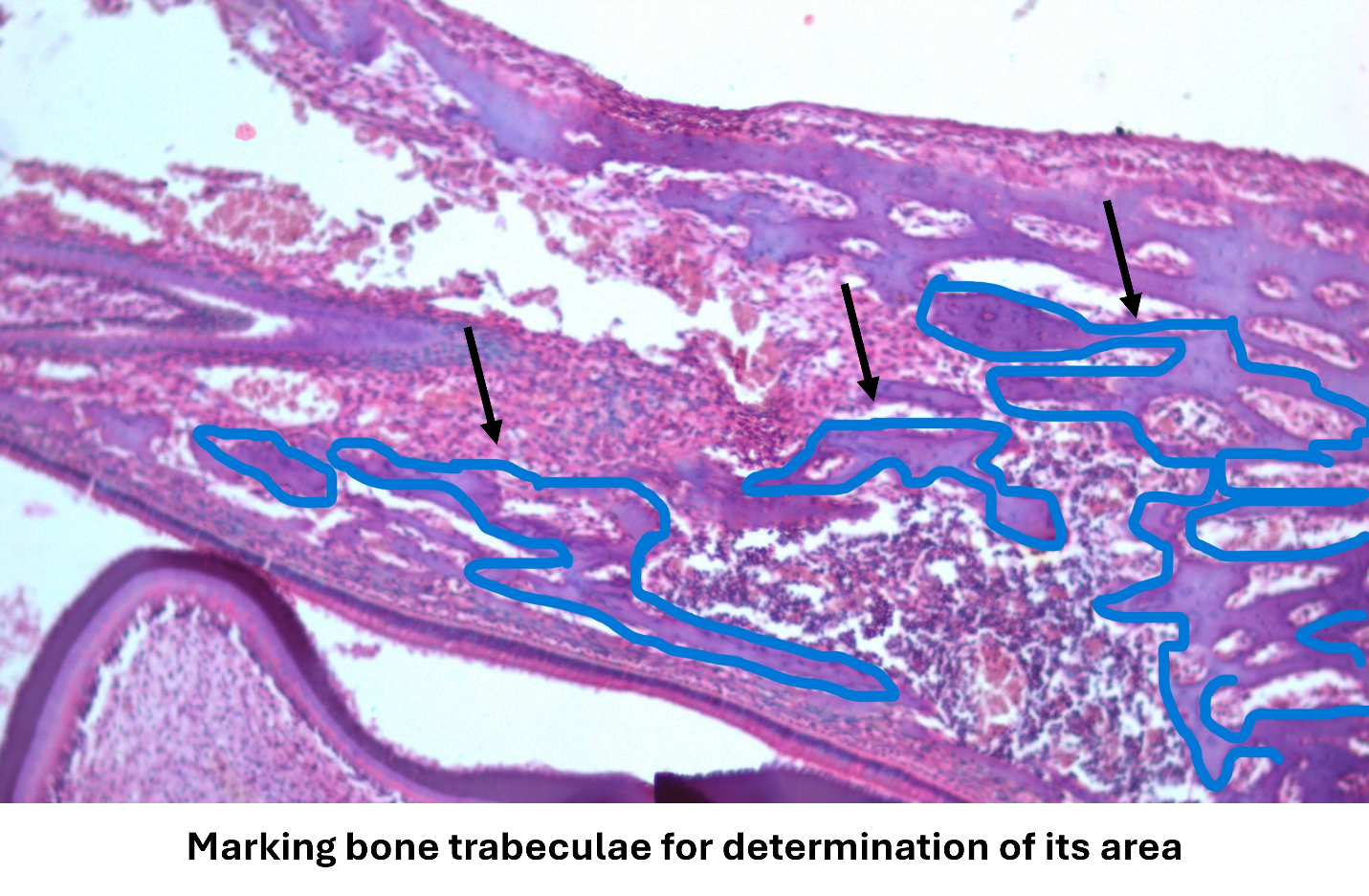


**Fig.S1:** A photomicrograph showing marking bone trabeculae for determination of its total area using image analysis software.


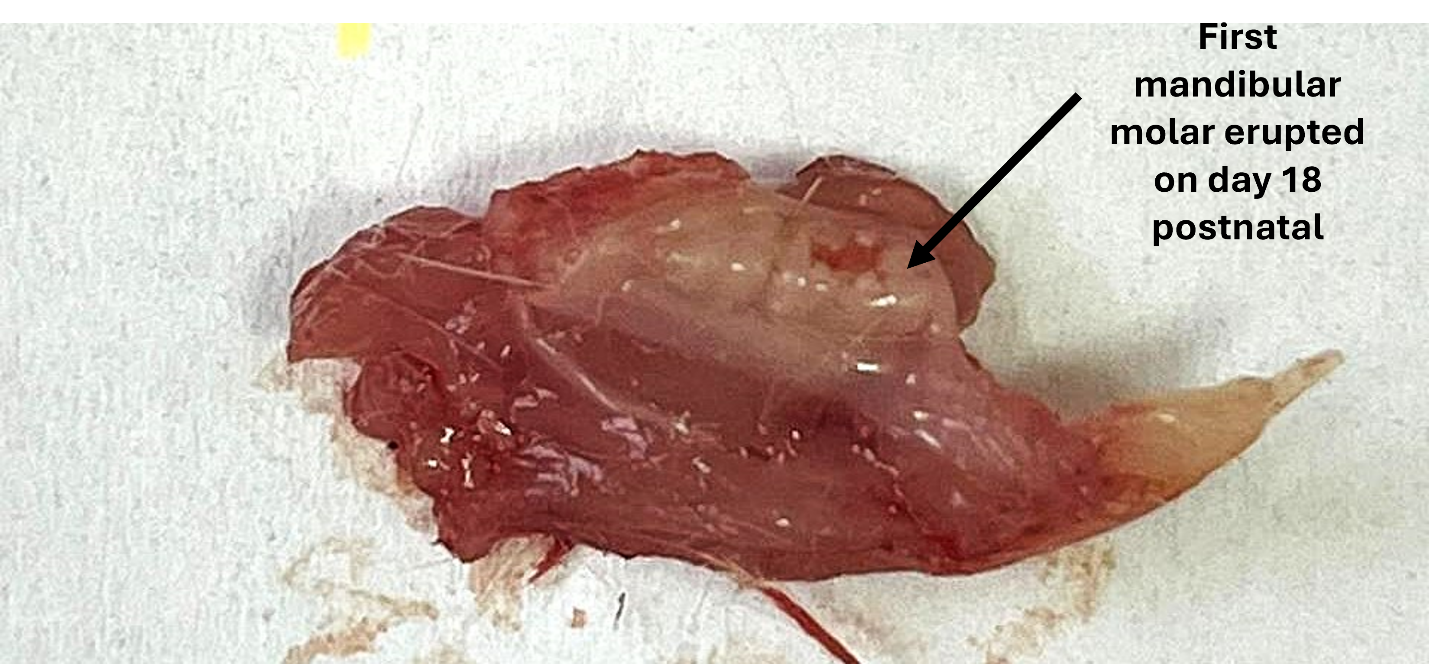


**Fig.S2:** A photograph showing the hemi-mandible of a pup on day 18 postnatal with the first mandibular molar completely erupted.
